# Clinical characteristics and prognostic impact of atrial fibrillation among older patients with heart failure with preserved ejection fraction hospitalized for acute heart failure

**DOI:** 10.1007/s11739-024-03754-w

**Published:** 2024-09-03

**Authors:** Giuseppe De Matteis, Maria Livia Burzo, Amato Serra, Davide Antonio Della Polla, Maria Anna Nicolazzi, Benedetta Simeoni, Antonio Gasbarrini, Francesco Franceschi, Giovanni Gambassi, Marcello Covino

**Affiliations:** 1 Department of Internal Medicine, Fondazione Policlinico Universitario A.Gemelli IRCCS, Rome, Italy; 2 Division of Internal Medicine, Ospedale Santo Spirito in Sassia, Rome, Italy; 3 Emergency Department, Fondazione Policlinico Universitario A.Gemelli IRCCS, Rome, Italy; 4 Università Cattolica del Sacro Cuore, Rome, Italy; 5 Department of Medicine and Translational Surgery, Università Cattolica del Sacro Cuore, Rome, Italy

**Keywords:** Atrial fibrillation, Heart failure with preserved ejection fraction, Acute heart failure, Mortality, Older patients

## Abstract

Heart failure with preserved ejection fraction (HFpEF) and atrial fibrillation (AF) are often coexisting conditions, but their interrelationship has not yet been clarified. This study investigated the clinical characteristics and prognostic impact of AF among older patients with HFpEF hospitalized for acute HF (AHF). The study included patients 65 years of age and older who were admitted to the Emergency Department due to AHF from 1 January 2016 to 31 December 2019. Patients were divided into two groups according to the presence of AF. The primary endpoint was all-cause, in-hospital mortality. Overall, 770 patients with HFpEF were included, mean age 82 years, 53% were females. Nearly, a third (30%) of these patients had a concomitant AF and they were significantly older and had higher N-Terminal pro-B-type natriuretic peptide (NT-proBNP) values. Overall, the in-hospital mortality rate was much higher among HFpEF patients with AF compared to those without AF (11.4% vs 6.9%, respectively; *p* = 0.037). At multivariate analysis, AF emerged as an independent risk factor for death (OR 1.73 [1.03–2.92]; *p* = 0.038). Among older patients with HFpEF admitted for AHF, the coexistence of AF was associated with a nearly twofold increased risk of all-cause in-hospital mortality. Patients with HFpEF and AF describe a phenotype of older and more symptomatic patients, with higher NT-proBNP, left atrial enlargement, right ventricular dysfunction, and higher CV mortality.

**Supplementary Information:**

The online version contains supplementary material available at 10.1007/s11739-024-03754-w.

## Introduction

Heart failure (HF) with preserved ejection fraction (HFpEF) is a major global health problem, accounting for approximately half of all HF cases [1]. HFpEF is a very complex and heterogeneous syndrome mostly involving females, often associated with older age and multiple comorbidities [2]. Although HFpEF prevalence is on the rise and it is associated with a risk of mortality and hospitalization as high as that of patients with heart failure with reduced ejection fraction (HFrEF)[3–5], the prognostic role of comorbidities remains not completely understood.

Atrial fibrillation (AF) is the most common arrhythmia in HF, occurring in at least one-third of all HFpEF patients, and is particularly prevalent in the aging phenotype [6–8]. A coexisting AF exerts a profound functional impact in HFpEF, a conditions which is already characterized by elevated LV filling pressure. Hemodynamic changes resulting from AF, such as impaired left ventricular (LV) filling and increased pulmonary wedge pressure, are clinically relevant in HFpEF, and contribute to further reduce cardiac output and worsening symptoms [9].

To date, studies that have attempted to clarify the prognostic role of AF in HFpEF have yielded contrasting results. On one hand, there appears to be evidence of an increased risk of hospitalization and mortality among HF patients with AF [6, 9, 10] with a greater impact among HFpEF patients compared to those with HFrEF [11]. On the other hand, other studies have failed to demonstrate a prognostic role for AF [5, 10].

Thus, this study aimed to investigate the clinical characteristics and prognostic impact of AF among older patients with HFpEF hospitalized for acute heart failure (AHF).

## Materials and methods

This is a single-center, retrospective study conducted in a large academic medical center, Fondazione Policlinico Universitario A. Gemelli IRCCS (Rome, Italy).

### Study population

We identified all patients 65 years of age and older consecutively admitted to the Emergency Department (ED) due to AHF and hospitalized in internal medicine wards, over a 4-year period between January 1, 2016, and December 31, 2019. We excluded data from 2020 onwards to avoid potential confounding factors related to the COVID-19 pandemic.

The criteria for identifying cases included an admission diagnosis of AHF, either de novo or acutely worsening HF, adjudicated by the emergency physician and based on a set of standardized parameters including clinical symptoms, physical examination, laboratory parameters, biomarkers, and radiological findings. In addition, cases needed to have AHF coded as the primary diagnosis in the discharge record. Diagnoses at hospital discharge were based on ICD-10 codes [International Classification of Disease, 10th revision].

Among these patients, we evaluated only those who underwent a 12-derivation electrocardiogram (EKG) and a cardiac ultrasound during the initial hospital stay. In the final sample, we included all patients classified as HFpEF—ejection fraction (EF) ≥ 50%—according to the European Society of Cardiology (ESC) guidelines [12] [Figure S1]. The diagnosis of AF was adjudicated according to the clinical history of AF and the EKG rhythm at enrollment. The final sample was divided into two groups: HFpEF patients “with AF” and “without AF”. In the patients “with AF” group we included patients with permanent, persistent, and paroxysmal AF.

Patients presenting to the ED with AHF due to acute coronary syndromes and requiring catheter-based interventions, those with advanced atrioventricular blocks or cardiac tamponade, patients with pacemakers or implantable cardioverter defibrillator, and those who were otherwise admitted to an intensive care unit (ICU), were excluded from the study.

### Study variables

Data were obtained from electronic medical records. Each patient’s record was used to collect demographics and clinical characteristics, data regarding ED presentation, as well as any information related to hospital stay, including diagnostic tests and procedures, treatments, and outcome. To gain further information, relevant medical documentation was reviewed to reach a complete account of all comorbidities.

The data considered in the study included:

- Demographic data: age and sex.
- Clinical presentation at ED admission, including vital signs [blood pressure, heart rate, oxygen saturation], body mass index (BMI), clinical symptoms (dyspnea, chest pain, syncope, fatigue), and physical signs such as the presence of peripheral edemas and oliguria. The New York Heart Association (NYHA) classification was used to categorize all patients according to the severity of HF symptoms [13].
- Echocardiographic variables, including LVEF, evaluated with conventional 2D-trans-thoracic echocardiogram according to international standard criteria [14], left ventricular end-systolic diameter (LVSd), left ventricular end-diastolic diameter (LVDd), tricuspid annular plane systolic excursion (TAPSE), pulmonary artery systolic pressure (PASP), E/e’ ratio and left atrial dimension index (LAVI).
- Laboratory parameters and biomarkers, including blood N-terminal pro-B-type natriuretic peptide (NT-proBNP), high-sensitivity cardiac troponin I (hs-cTnI), hemoglobin (Hb), white blood cell count (WBC), platelet count (PLT), creatinine, glucose levels, procalcitonin and C-reactive protein (CRP).
- Comorbidities, including hypertension, ischemic heart disease (IHD), peripheral artery disease (PAD), cerebrovascular disease (history of previous stroke), dementia, chronic obstructive pulmonary disease (COPD), diabetes, chronic kidney disease (CKD) and malignancy. The number of comorbidities and their severity were assessed by the Charlson Comorbidity Index (CCI) [15].
- Medications prescribed at hospital discharge, including loop diuretics, beta-blockers, mineralocorticoid antagonists (MRA), angiotensin-converting enzyme inhibitors (ACEi), angiotensin receptor blockers (ARB), antiarrhythmic and anticoagulants (including both oral and parenteral anticoagulants).

### Outcome measures

The primary endpoint of the study was the all-cause, in-hospital mortality. In addition, we analyzed separately the occurrence of cardiovascular (CV)- and non-CV-related deaths.

Based on electronic health records and on the hospital-based death certificates, the causes of death were distinguished between CV- and non-CV death. CV death events were defined as deaths occurring due to terminal HF and cardiogenic shock, acute myocardial infarction, arrhythmias, acute pulmonary embolism, cardiac tamponade, and acute cerebrovascular disease. Non-CV-related events were defined as deaths occurring due to respiratory failure, severe sepsis/septic shock, renal failure, and to bleeding with hemorrhagic shock.

The secondary endpoint was the length of hospital stay (LOS), calculated as the time from ED admission to hospital discharge or death.

### Statistical analysis

Categorical variables were presented as numbers and percentages. Continuous normally distributed variables were presented as mean ± standard deviation, non-normally distributed data were presented as median (inter-quartile range), and binary or ordinal variables were presented as absolute frequency (%). Parametric variables were compared by the Mann–Whitney *U* test, whereas categorical variables were compared by the Chi-square test (with Fisher test if indicated). Significant variables at univariate analysis were entered into a multivariate logistic regression model to identify independent predictors for the outcomes. To avoid overfitting and overestimation of the parameters, the variables with high collinearity were excluded from the multivariate models. If possible, categorical variables were preferred to continuous. The single items composing cumulative variables (i.e., Charlson index) were excluded from the model to avoid redundancy. The results of the logistic regression analysis are reported as odds ratio (OR) (95% confidence interval). Survival analysis was performed according to the Kaplan–Meier approach.

All data were analyzed by SPSS v26® (IBM, NY, USA). A two-sided *p* value of 0.05 or less was considered statistically significant.

### Statement of ethics

The investigation conforms to the principles outlined in the Declaration of Helsinki and was approved by the local ethical committee (IRB #0051814/19).

## Results

### Study cohort and baseline characteristics

Overall, 770 patients were included in the study. Baseline characteristics are illustrated in Table 1. The median age was 82 years [75–87 years] and females accounted for more than 50% of the patients (53%). About one-third (30%) of patients had a confirmed diagnosis of AF. Among them, 36 (15.3%) cases were classified as paroxysmal AF and 180 (76.3%) cases as permanent and persistent AF. No sufficient data were available to determine the type of AF in 20 patients (8.4%).

**Table 1:** Baseline characteristics of patients with HFpEF admitted to ED for AHF stratified by coexisting atrial fibrillation^§^

	All n 770	HFpEF without AF n 534	HFpEF with AF n 236	*p* value
Age	82 [75–87]	81 [74–87]	83 [77–88]	0.004
Sex (male)	360 (46.8%)	264 (49.4%)	96 (40.7%)	0.025
Emergency department presentation				
NYHA class				
(I–II)	147 (19.1%)	97 (18.2%)	50 (21.2%)	
(III)	510 (66.2%)	358 (67%)	152 (64.4%)	0.452
(IV)	113 (14.7%)	79 (14.8%)	34 (14.4%)	
Dyspnea	404 (52.5%)	264 (49.4%)	140 (59.3%)	0.011
Chest pain	125 (16.2%)	96 (18%)	29 (12.3%)	0.048
Syncope	53 (6.9%)	47 (8.8%)	6 (2.5%)	0.002
Peripheral edemas	172 (22.3)	119 (22.3%)	53 (22.5%)	0.958
Fever	138 (17.8%)	90 (16.9%)	48 (19.8%)	0.182
Fatigue	93 (12.1%)	65 (12.2%)	28 (11.9%)	0.904
Oliguria	19 (2.5%)	13 (2.4%)	6 (2.5%)	0.929
Body mass index	26.5 [23.6–29.9]	26.3 [23.6–29.7]	27.5 [23.6–30.4]	0.097
Heart rate (BPM)	82 [70–97]	81 [69–96]	84 [72–98]	0.090
Systolic blood pressure (mmHg)	130 [112–150]	131 [115–153]	130 [110–146]	0.052
Diastolic blood pressure (mmHg)	73 [61–83]	72 [62–82]	73 [61–85]	0.386
Peripheral oxygen saturation (%)	95 [91–97]	95 [92–98]	95 [91–97]	0.349
Echocardiographic findings				
LVEF (%)	58 [55–62]	59 [55–63]	57 [54–60]	< 0.001
Left ventricular hypertrophy	263 (71.1%)	180 (74.4%)	83 (64.8%)	0.036
LVDd (mm)	47.0 [42.0–52.0]	48.0 [41.5–52.0]	47.0 [43.0–52.0]	0.834
LVSd (mm)	30.0 [26.0–35.0]	30.0 [26.0–35.0]	31.0 [26.0–35.0]	0.433
TAPSE (mm)	19 [17–23]	20 [17–23]	18 [16–19]	< 0.001
PASP (mmHg)	40 [32–50]	40 [30–46]	40 [35–50]	0.002
E/e’	10 [8–15]	11 [9–15]	15 [10–16]	0.027
LAVI (mL/m^2^)	56 [47–62]	55 [46–61]	59 [53–64]	< 0.001
Left ventricular diastolic dysfunction (LVDD) ultrasound classification				
Normal	27 (14.7%)	22 (13.4%)	5 (25.0%)	
Grade 1	39 (21.2%)	37 (22.6%)	2 (10.0%)	0.251
Grade 2	102 (55.4%)	92 (56.1%)	10 (50.0%)	
Grade 3	16 (8.7%)	13 (7.9%)	3 (15.0%)	
Laboratory findings				
Hb (gr/dL)	11.0 [9.8–12.5]	11.1 [9.7–12.5]	11.0 [10.0–12.4]	0.991
WBC (× 10^9^)	9.1 [6.8–12.7]	9.1 [6.8–12.3]	9.05 [6.8–13.0]	0.621
PLT (× 10^9^/L)	233 [171–311]	233 [270–311]	236 [193–309]	0.710
Creatinine (mg/dL)	1.2 [0.88–1.95]	1.3 [0.88–2.07]	1.2 [0.92–1.77]	0.981
Glucose (mg/dl)	127 [103–164]	128 [101–166]	126 [105–163]	0.712
NT-proBNP (pg/mL)	5473 [2230–13321]	4749 [1994–13180]	6321 [3354–13398]	< 0.001
u-hscTnI (pg/mL)	0.05 [0.29–0.82]	0.26 [0.05–0.81]	0.35 [0.08–0.89]	0.094
Procalcitonin (ng/mL)	0.08 [0.05–0.41]	0.09 [0.05–0.23]	0.07 [0.05–0.72]	0.913
C-reactive protein (mg/L)	31.2 [11.1–92.7]	40.4 [10.9–91.8]	14.8 [11.6–40.8]	0.103
Comorbidities				
CCI	6 [5–8]	6 [5–8]	6 [5–8]	0.637
Hypertension	427 (55.5%)	301 (56.4%)	126 (53.4%)	0.443
Ischemic heart disease	170 (22.1%)	125 (23.4%)	45 (19.1%)	0.101
PAD	109 (14.2%)	83 (15.5%)	26 (11%)	0.060
Cerebrovascular disease	45 (5.8%)	29 (5.4%)	16 (6.8%)	0.280
Dementia	39 (5.1%)	20 (3.7%)	19 (8.1%)	0.120
COPD	179 (23.2%)	112 (21%)	67 (28.4%)	0.022
Diabetes	186 (24.2%)	131 (24.5%)	55 (23.3%)	0.714
Chronic kidney disease	218 (28.3%)	151 (28.3%)	67 (28.4%)	0.511
Malignancy	82 (10.6%)	64 (12%)	18 (7.4%)	0.035
Medications				
Loop diuretics	515 (85.5%)	334 (82.5%)	181 (91.9%)	0.001
Beta-blockers	458 (76.1%)	300 (74.1%)	158 (80.2%)	0.059
Mineralocorticoid antagonists	315 (52.3%)	203 (50.1%)	112 (56.9%)	0.071
ACE inhibitors	160 (26.6%)	114 (28.1%)	46 (23.4%)	0.124
ARB	122 (20.3%)	87 (21.5%)	35 (17.8%)	0.170
Antiarrhythmic drugs	11 (1.8%)	0 (0%)	11 (5.2%)	< 0.001
Anticoagulants	214 (27.7%)	27 (5%)	187 (87.7%)	< 0.001
Outcomes				
LOS (days)	9.5 [5.5–15.3]	9.5 [5.5–15]	9.5 [5–15]	0.678
Cardiovascular death	64 (8.2%)	37 (6.9%)	27 (11.2%)	0.035
Non-cardiovascular death	45 (5.8%)	26 (4.9%)	19 (7.9%)	0.072

^§^We included in the AF group both patients with paroxysmal AF and permanent AF

*ACE* angiotensin-converting enzyme, *AF* atrial fibrillation; *AHF* Acute heart failure, *ARB* angiotensin receptor blockers, *BPM* beats per minute, *CCI* Charlson comorbidity Index, *COPD* chronic obstructive pulmonary disease, *ED* emergency department, Hb, hemoglobin, *HFpEF* heart failure with preserved ejection fraction, *hs-cTnI* high-sensitivity cardiac troponin I, *LOS* length of stay, *LVEF* left ventricular ejection fraction, *LVDd* left ventricular end-diastolic diameter; *LVSd* left ventricular end-systolic diameter, *LAVI* left atrial dimension index, *NYHA* New York Heart Association, *NT-proBNP* N-terminal pro-B-type natriuretic peptide, *PAD* peripheral artery disease, *PASP* pulmonary artery systolic pressure, *PLT* platelets, *TAPSE* tricuspid annular plane systolic excursion, *WBC* white blood cell

Patients with AF were significantly older compared with those without AF and even more commonly females. Dyspnea was the prevailing symptom at ED presentation (53%) followed by peripheral edema (22%) and chest pain (16%). At ED presentations, two-thirds of patients (66%) were classified as NYHA class III, while 19% were on class I or II. No differences in NYHA class were found among patients with AF and without AF.

HFpEF patients with AF displayed lower EF (57% vs. 59%) and a higher NT-proBNP median value (6321 pg/mL vs. 4749 pg/mL). As a result of the analysis of echocardiographic data, we found that 71.1% of the whole sample was affected by left ventricular hypertrophy, and it was more represented in the non-AF group (74.4% vs 64.8%). Furthermore, HFpEF patients with AF showed higher median values of E/e’ ratio (15 vs 11) and lower median values of TAPSE (18 mm vs 20 mm) than those without AF. As expected, patients with AF had significantly increased right atrium volumes, expressed by the LAVI (59 mL/m^2^ vs 55 mL/m^2^).

Overall, HFpEF patients either with or without AF had a high burden of comorbidities, with a median CCI of 6. Over half of patients had hypertension, 25% had a diagnosis of diabetes and 28% had CKD. Among HFpEF patients with AF, a history of cerebrovascular diseases (7% vs 5%) and a diagnosis of dementia (8% vs 4%) was more prevalent. Conversely, the prevalence of COPD was significantly higher in patients with AF (28% vs 21%).

Information about pharmacological treatment was available for 602 patients (about 78% of the sample). Loop diuretics were the most common class of medications (85.5% of the whole sample), and they were significantly more prescribed in the AF patients’ group (91.9% vs 82.5%). Among the other medications recommended for HF by clinical guidelines, such as ACE inhibitors, ARB, MRA, and beta-blockers, no statistically significant differences were found between the two study groups. As expected, anticoagulant use was common in most patients with HFpEF and AF (87.7%). Furthermore, the use of antiarrhythmic medications was reported in a relatively small percentage of AF patients (5.2%).

Overall, the median LOS was 9.5 days and did not differ between the two groups.

The mortality rate due to CV causes was significantly higher in patients with AF than in patients without AF (11% vs. 6.9%), while no significant differences in non-CV death were reported between the two groups (7.9% vs 4.9%).

### Variables associated with the primary endpoint (survival analysis)

Over the observed period, 64 patients (8%) died. As illustrated in Table 2, deceased patients were significantly older (84 vs 81 years), presenting with more severe symptoms as expressed also by NYHA class III (80% vs 65%), lower systolic BP (121 mmHg vs 131 mmHg) and reduced peripheral oxygen saturation (93% vs 95%).

**Table 2:** Univariate and multivariate analyses with respect to all-cause in-hospital death in HFpEF patients admitted to ED with AHF and subsequently hospitalized

	Survived n 706	Deceased n 64	Univariate analysis *p* value	Odds ratio	Multivariate analysis *p* value
Age	81 [75–87]	84 [79–88]	0.036	1.03 [0.99–1.07]	0.077
Sex (male)	328 (46.5%)	32 (50%)	0.587		
Emergency department presentation					
NYHA class					
(I–II)	143 (20.3%)	4 (6.3%)		Reference	
(III)	459 (65%)	51 (79.7%)	0.019	0.53 [0.13–2.01]	0.349
(IV)	104 (14.7%)	9 (14.1%)		1.83 [0.74–4.47]	0.186
Dyspnea	364 (51.6%)	40 (62.5%)	0.093		
Chest pain	117 (16.6%)	8 (12.5%)	0.398		
Syncope	51 (7.2%)	2 (3.1%)	0.215		
Peripheral edemas	162 (22.9%)	10 (15.6%)	0.178		
Fatigue	86 (12.2%)	7 (10.9%)	0.770		
Fever	125 (17.6%)	13 (20.3%)	0.342		
Oliguria	19 (2.7%)	0 (0%)	0.184		
Body mass index	26.5 [23.6–30.1]	26.6 [24.3–29.4]	0.895		
Heart rate (BPM)	82 [70—97]	82 [75—100]	0.298		
Systolic blood pressure (mmHg)	131 [113—152]	121 [106—140]	0.002	0.98 [0.97–0.99]	0.001
Diastolic blood pressure (mmHg)	73 [62—84]	70 [60—80]	0.086		
Peripheral oxygen saturation (%)	95 [92—98]	93 [89—96]	0.006	0.96 [0.92–1.05]	0.166
Echocardiographic findings					
LVEF (%)	58 [55–62]	57 [55–62]	0.609		
Left ventricular hypertrophy	241 (71.5%)	22 (66.7%)	0.343		
LVDd (mm)	48 [42–52]	47 [42–50]	0.566		
LVSd (mm)	31 [26–35]	31 [24–33]	0.457		
TAPSE (mm)	19 [17–22]	18 [15–21]	0.150		
PASP (mmHg)	40 [32–49]	45 [35–61]	0.014		
E/e’	12 [9–15]	11 [8–15]	0.624		
LAVI (mL/m^2^)	57 [48–62]	60 [46–63]	0.589		
Left ventricular diastolic dysfunction (LVDD) ultrasound classification					
Normal	24 (14.1%)	3 (21.4%)			
Grade 1	37 (21.8%)	2 (14.3%)	0.316		
Grade 2	93 (54.7%)	9 (64.3%)			
Grade 3	16 (9.4%)	0 (0%)			
*Laboratory findings*					
Hb (gr/dL)	11.3 [9.9–12.9]	11.0 [9.8–12.5]	0.35		
WBC (× 10^9^)	8.5 [6.8–10.7]	10.7 [7.8–12.6]	0.600		
PLT (× 10^9^/L)	234 [181–310]	222 [186–350]	0.723		
Creatinine (mg/dL)	1.24 [0.89–1.90]	1.55 [1.04–2.53]	0.006	0.88 [0.65–1.17]	0.386
Glucose (mg/dl)	128 [102–165]	122 [100–162]	0.715		
NT-proBNP (pg/ml)	3789 [1818–8748]	8632 [5357–27884]	< 0.001	1.04 [1.02–1.06]^§^	< 0.001
u-hscTnI (pg/mL)	0.28 [0.05–0.08]	0.44 [0.14–1.23]	0.002	1.62 [1.17–2.22]	0.003
Procalcitonin (ng/mL)	0.080 [0.05–0.52]	0.16 [0.05–0.23]	0.770		
C-reactive protein (mg/dL)	20.9 [11.1–72.1]	83.5 [22.3–200.6]	0.042		
Comorbidities					
CCI	6 [5–8]	7 [6–9]	< 0.001	1.30 [1.14–1.51]	< 0.001
Hypertension	392 (55.5%)	35 (54.7%)	0.897		
Ischemic heart disease	148 (21%)	22 (34.4%)	0.013		
PAD	94 (13.3%)	15 (23.4%)	0.026		
Cerebrovascular disease	38 (5.4%)	7 (11%)	0.070		
Dementia	33 (4.7%)	6 (9.4%)	0.090		
COPD	157 (22.2%)	22 (34.4%)	0.020		
Diabetes	162 (22.9%)	24 (37.5%)	0.009		
Chronic kidney disease	186 (26.3%)	32 (50%)	< 0.001		
Atrial fibrillation	209 (29.6%)	27 (42.2%)	0.037	1.73 [1.03–2.92]	0.038
Malignancy	76 (10.7%)	6 (9.4%)	0.474		
LOS (days)	9.5 [5.9–15]	10.5 [3–18]	0.700		

^§^Odds ratios are calculated for each 1000-fold increase in NT-proBNP

*AF* atrial fibrillation, *AHF* acute heart failure, *BPM* beats per minute, *CCI* Charlson comorbidity Index, *COPD* chronic obstructive pulmonary disease, *ED* emergency department, *Hb* hemoglobin, *HFpEF* heart failure with preserved ejection fraction, *hs-cTnI* high-sensitivity cardiac troponin I, *LOS* length of stay, *LVEF* left ventricular ejection fraction, *LVDd* left ventricular end-diastolic diameter, *LVSd* left ventricular end-systolic diameter, *LAVI* left atrial dimension index *NYHA* New York Heart Association, *NT-proBNP* N-terminal pro-B-type natriuretic peptide, *PAD* peripheral artery disease, *PASP* pulmonary artery systolic pressure, *PLT* platelets, *TAPSE* tricuspid annular plane systolic excursion, *WBC* white blood cell

A concomitant diagnosis of AF was more prevalent among patients who died (42% vs 30%). Laboratory tests for patients who died documented higher NT-proBNP, u-hscTnI, and creatinine median values.

Deceased patients also had a higher number of comorbidities (CCI = 7), primarily hypertension and CKD. No statistically significant differences were found in the prevalence of each comorbidity between deceased and surviving patients.

### Multivariate analysis for in-hospital death

**Fig. 1:**
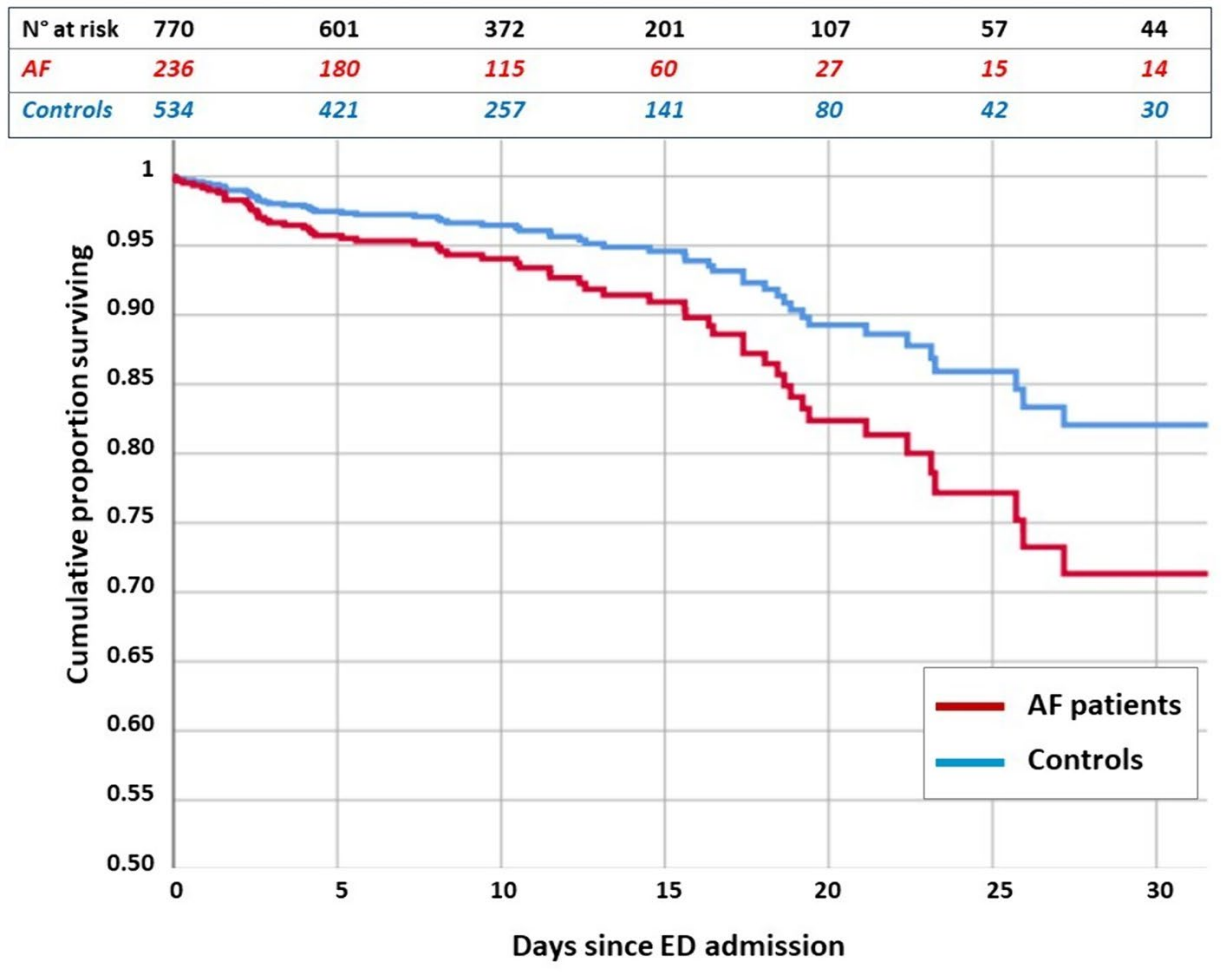
Cumulative proportion of survival in patients with HFpEF hospitalized for AHF with and without AF. Once corrected for clinical characteristic and comorbidities, AF emerged as an independent risk factor for all-cause, in-hospital death (OR 1.73 [1.03–2.92]; *p* = 0.038). *AF* atrial fibrillation, *AHF* acute heart failure, *HFpEF* heart failure with preserved ejection fraction

In the multivariate Cox regression analysis, AF emerged as an independent risk factor for all-cause, in-hospital death (OR 1.73 [1.03–2.92]; *p* = 0.038) [Table 2, Fig. 1]. Similarly, a high number of comorbidities was associated with an increased risk of mortality (OR 1.30 [1.14–1.51]; < 0.001).

Values of NT-proBNP and u-hscTnI above the median were also found associated with mortality (OR 1.04 [1.02–1.06]; *p* < 0.001 and OR 1.62 [1.17–2.22; *p* = 0.003], respectively).

## Discussion

Our study has documented that among older patients with HFpEF admitted for AHF, the concomitant presence of AF was associated with a nearly two-fold increased risk of all-cause in-hospital mortality. Patients with HFpEF and AF describe a phenotype of older and more symptomatic patients, with higher NT-proBNP, left atrial enlargement, right ventricular dysfunction, and higher CV mortality.

HFpEF is a global health problem that is gradually emerging as the predominant form of HF. Patients with HFpEF tend to be older, and multiple coexisting conditions define both their symptoms and outcomes [9, 16]. Among coexisting conditions, AF is common and likely underdiagnosed in HFpEF [17]. Due to the great heterogeneity of HFpEF patients, it is of paramount importance identifying the correlates associated with worse outcomes. In this respect, the impact of AF on in-hospital outcomes remains a matter of debate.

In the present study, AF was found in one-third of patients, and they were significantly older, most commonly women, presented with more symptoms, and with significantly higher NT-proBNP than patients without AF. These characteristics align with one of the described phenotypes of HFpEF patients—mainly older patients with coexisting AF, higher degrees of comorbidity, and elevated NT-proBNP —that has been associated with a worse prognosis, and an increased risk of hospitalizations for AHF [1, 5, 10, 18]. Moreover, as expected, patients with HFpEF and concomitant AF had higher LAVI values, being AF already associated with higher LAVI and left atrial remodeling in HFpEF [19]. In addition, a significantly lower TAPSE was reported in patients with AF. In previous reports Gorter et al. highlighted that patients with AF and HFpEF displayed more right ventricular dysfunction than patients without any history of AF, and that right ventricular dysfunction and AF are common in patients with HFpEF, often coexisting and independently associated with a worse prognosis [20, 21].

Furthermore, among patients with HFpEF and AF we outlined an increased CV mortality. As recently described, the presence of AF in HFpEF is associated with cardiac structural and functional changes together with altered expression of several fibro-inflammatory biomarkers [22]. In addition, in a latest analysis, Saksena et al. described that AF is strongly associated not only with risk of HF and CV hospitalization, but also with symptomatic HF progression in early stages of HFpEF and increased pump mortality in advanced symptomatic HFpEF [23].

The main finding of this study is that among acutely decompensated older patients with HFpEF, AF was associated with a 1.7 increased risk of all-cause mortality. A previously published large meta-analysis of several studies including HFpEF patients has found that AF was associated with an 11% increased risk of all-cause mortality [9]. Similarly, a retrospective single-center study showed not only that AF is common in the setting of AHF and is associated with an overall 1.8 increased risk of all-cause mortality, but also that this association is present only in patients with HFpEF and not in patients with HFrEF [24]. Likewise, a study based on patients from the National Inpatient Sample has analyzed hospitalizations for HFpEF and HFrEF, with and without AF, highlighting an adverse impact of AF only on those with HFpEF [25]. In addition, a post-hoc analysis of the TOPCAT (Treatment of Preserved Cardiac Function Heart Failure With an Aldosterone Antagonist) trial has shown that AF was associated with a 2.5-fold increase in all-cause mortality [6].

The observed increase in mortality among HFpEF patients with AF could be due to several factors. HFpEF is characterized by impaired relaxation and decreased ventricular compliance, leading to increased atrial filling pressure. This causes atrial fibrosis and left atrial myopathy, which are arrhythmogenic and promote AF. AF results in the loss of atrial systole and chronotropic dysregulation, reducing diastolic filling time and left ventricular filling, impairing cardiac reserve and worsening symptoms [26]. Consequently, HFpEF patients with AF exhibit chronotropic incompetence and exercise intolerance. In addition, compared to HFpEF patients in sinus rhythm, those with AF have lower peak oxygen consumption, higher mean pulmonary capillary wedge pressure, and worse diastolic and right ventricular function [19] [21]. In addition, atrial fibrillation increases the incidence of fatal thromboembolic events, contributing to nearly 20–30% of all ischemic strokes and 10% of cryptogenic strokes [27].

In the present analysis, we also showed that among older HFpEF patients hospitalized with AHF, higher NT-proBNP values at ED presentation were associated with an increased risk of mortality. The magnitude of NT-proBNP elevation has been consistently associated with adverse outcomes in the general population and in patients with HF across the EF spectrum [28]. Among HFpEF patients, higher NT-proBNP values were associated with significantly worse LV stiffness and left atrial function compared with patients with lower NT-proBNP [29]. Consistently, a correlation between elevated NT-proBNP values and an increase in mortality has been demonstrated among patients with HFpEF [30, 31].

A higher number of comorbidities was also associated with an increase in mortality. HFpEF patients have a larger number of concomitant diseases than patients with HFrEF in both sexes [32] [33] and the presence of comorbidities has been shown to exert a substantial influence on the prognosis of HFpEF patients [34, 35].

## Limitations

Some limitations of our study warrant consideration. First, due to its retrospective nature, all diagnoses and related classification of cases were based on the information available in the hospital-based, electronic medical records. As a result, there may have been misclassifications of some patients. Second, this is a single-center study, therefore, the enrolled patients could not be representative of the entire HFpEF population. Third, we did not consider the duration of AF when co-diagnosed with HFpEF, therefore, we cannot infer if the temporal relationship may have influenced our result. Finally, no complete information was available about the pharmacological treatment during hospitalization. In this respect, it is noteworthy that patients were hospitalized in internal medicine wards with permanent medical staff under the same coordinating chief, constantly reevaluating and implementing clinical guidelines on treatment. Due to the enrollment deadline, it was impossible to detect the data about the use of glyphozines, introduced in HFpEF treatment until 2023 [36]. The specific causes of death were derived from death certificates that may not have always been accurate.

## Conclusions

Among older patients with HFpEF admitted for AHF, the coexistence of AF was associated with a nearly twofold increased risk for all-cause mortality.

Patients with HFpEF and AF described a phenotype of older and more symptomatic patients, with higher NT-proBNP, left atrial enlargement, right ventricular dysfunction, and higher CV mortality.

Whether the observed increase in mortality is primarily related to AF or a consequence of the cardiac abnormalities leading to diastolic dysfunction, in which AF is merely a bystander, is a question that will need to be addressed in future research. However, an accurate and timely recognition of this high-risk phenotype, along with a tailored management of AF should be prioritized to prevent excess mortality. Further studies are needed to understand whether an approach to rate control alone or rhythm restoration is the most appropriate strategy to improve prognosis in these patients.

## Supplementary Information

Below is the link to the electronic supplementary material.

Supplementary file1 Fig. S1 Flow chart of the cohort selection for the study. *ICD* Implantable cardioverter defibrillator, *HFmrEF* Heart failure with midly reduces ejection fraction, *HFpEF* Heart failure with preserved ejection fraction, *HFrEF* Heart failure with reduced ejection fraction, *PMK* Pacemaker (JPG 60 KB)

## Data Availability

The datasets generated during and/or analyzed during the current study are available from the corresponding author on reasonable request.
