# Supplementary figures and images for: Clinical characteristics and prognostic impact of atrial fibrillation among older patients with heart failure with preserved ejection fraction hospitalized for acute heart failure
Source: Intern Emerg Med. 2024 Sep 3;20(1):95–104. doi: 10.1007/s11739-024-03754-w (PMC11794344; doi:10.1007/s11739-024-03754-w)

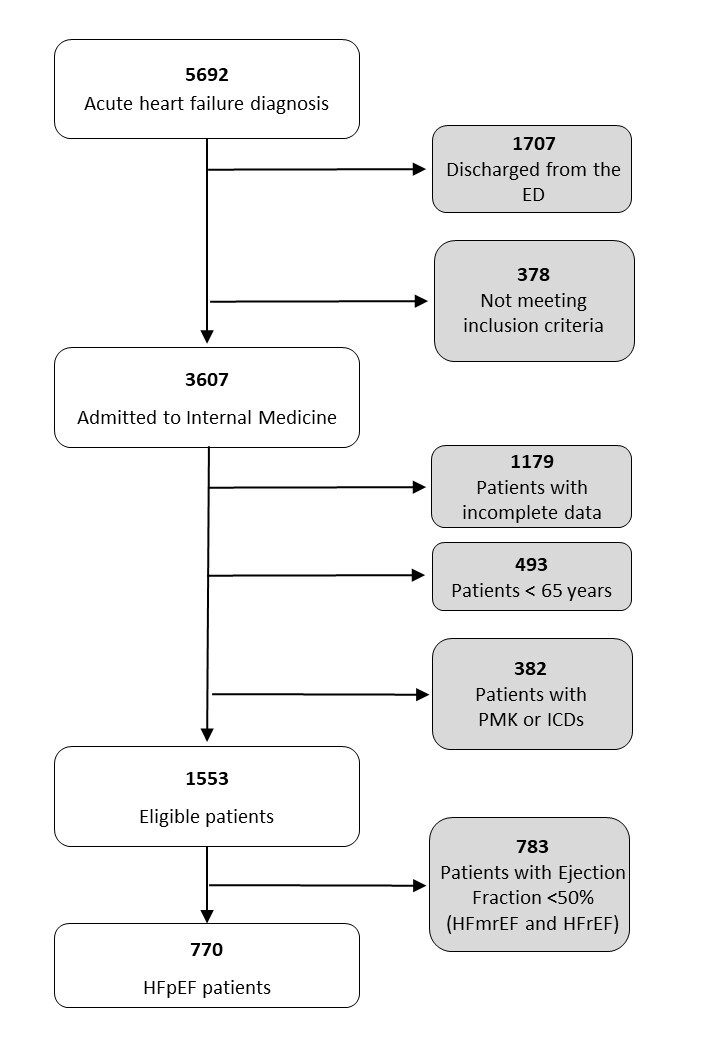

Supplement: Supplementary file 1 — Supplementary file1 Fig. S1 Flow chart of the cohort selection for the study. ICD Implantable cardioverter defibrillator, HFmrEF Heart failure with midly reduces ejection fraction, HFpEF Heart failure with preserved ejection fraction, HFrEF Heart failure with reduced ejection fraction, PMK Pacemaker (JPG 60 KB) [file 11739_2024_3754_MOESM1_ESM.jpg]
